# Unraveling Host-Vector-Arbovirus Interactions by Two-Gene High Resolution Melting Mosquito Bloodmeal Analysis in a Kenyan Wildlife-Livestock Interface

**DOI:** 10.1371/journal.pone.0134375

**Published:** 2015-07-31

**Authors:** David Omondi, Daniel K. Masiga, Yvonne Ukamaka Ajamma, Burtram C. Fielding, Laban Njoroge, Jandouwe Villinger

**Affiliations:** 1 Martin Lüscher Emerging Infectious Disease (ML-EID) Laboratory, International Centre for Insect Physiology and Ecology, P. O Box 30772-00100, Nairobi, Kenya; 2 Molecular Biology and Virology Laboratory, Department of Medical Biosciences, University of Western Cape, Private Bag X17, Bellville, 7535, South Africa; 3 Biochemistry and Molecular Biology Department, Egerton University, P.O Box 536, Egerton, 20115, Kenya; 4 Invertebrates Zoology Section, Zoology Department, National Museums of Kenya, P.O. Box 40658-00100, Nairobi, Kenya; University of Queensland &amp; CSIRO Biosecurity Flagship, AUSTRALIA

## Abstract

The blood-feeding patterns of mosquitoes are directly linked to the spread of pathogens that they transmit. Efficient identification of arthropod vector bloodmeal hosts can identify the diversity of vertebrate species potentially involved in disease transmission cycles. While molecular bloodmeal analyses rely on sequencing of cytochrome b (*cyt b*) or cytochrome oxidase 1 gene PCR products, recently developed bloodmeal host identification based on high resolution melting (HRM) analyses of *cyt b* PCR products is more cost-effective. To resolve the diverse vertebrate hosts that mosquitoes may potentially feed on in sub-Saharan Africa, we utilized HRM profiles of both *cyt b* and 16S ribosomal RNA genes. Among 445 blood-fed *Aedeomyia*, *Aedes*, *Anopheles*, *Culex*, *Mansonia*, and *Mimomyia* mosquitoes from Kenya’s Lake Victoria and Lake Baringo regions where many mosquito-transmitted pathogens are endemic, we identified 33 bloodmeal hosts including humans, eight domestic animal species, six peridomestic animal species and 18 wildlife species. This resolution of vertebrate host species was only possible by comparing profiles of both *cyt b* and 16S markers, as melting profiles of some pairs of species were similar for either marker but not both. We identified mixed bloodmeals in a *Culex pipiens* from Mbita that had fed on a goat and a human and in two *Mansonia africana* mosquitoes from Baringo that each had fed on a rodent (*Arvicanthis niloticus*) in addition to a human or baboon. We further detected Sindbis and Bunyamwera viruses in blood-fed mosquito homogenates by Vero cell culture and RT-PCR in *Culex*, *Aedeomyia*, *Anopheles* and *Mansonia* mosquitoes from Baringo that had fed on humans and livestock. The observed mosquito feeding on both arbovirus amplifying hosts (including sheep and goats) and possible arbovirus reservoirs (birds, porcupine, baboons, rodents) informs arbovirus disease epidemiology and vector control strategies.

## Introduction

Arthropod vectored pathogens contribute to the greatest diversity of neglected tropical diseases (NTDs) that significantly impact human health and livestock-based food security in developing countries and also threaten human and livestock health in developed countries [1]. Arthropod disease vectors may feed on a variety of vertebrate hosts, including wildlife that may represent unknown pathogen reservoirs, not only of known NTDs, but also of emerging infectious diseases (EIDs) [2]. Bloodmeal identification of field-collected vectors is pivotal to disentangling disease transmission dynamics, identifying ecological reservoirs during inter-epidemic periods, and developing appropriate disease control and response strategies [3]. However, most standard approaches to bloodmeal analysis rely on time consuming and expensive sequencing [4–6]. Peña et al. (2012) developed an improved, cost-effective and rapid approach based on high resolution melting analysis (HRM) of cytochrome b (*cyt b*) Polymerase Chain Reaction (PCR) products to identify vertebrate bloodmeals of triatomine bugs (*Rhodnius* and *Triatoma* species) in the Caribbean region of Colombia [7]. However, our preliminary analysis found that HRM analyses of these *cyt b* amplicons alone could not reliably differentiate the diversity of potential mosquito bloodmeal host species in East Africa, where humans, livestock and wildlife are often in close proximity.

To improve the vertebrate host species resolution of PCR-HRM based mosquito bloodmeal analysis in the East African context, we designed an additional pair of vertebrate specific PCR primers flanking short polymorphic mitochondrial 16S rRNA sequences amenable to robust HRM species typing. We used both *cyt b* [7] and 16S HRM analysis to resolve and identify mosquito blood-feeding sources found at the geographical interface of wildlife with livestock farming and of aquatic/wetland with terrestrial ecosystems along the shores and adjacent islands of Lakes Victoria and Baringo in Kenya. As both regions have documented recent arbovirus activity and support a diversity of vertebrate hosts that are critical to arthropod pathogen transmission [8–10], we also screened for arboviruses in the blood-fed mosquitoes.

## Materials and Methods

### Sampling areas

The study was carried out in Kenya, along the shores and adjacent islands of Lake Victoria in Homa Bay County and Lake Baringo in Baringo County. Before sampling, we obtained ethical clearance for the study from the Kenya Medical Research Institute (KEMRI) ethics review committee (Approval Ref: Non-SSC Protocol #310). Sampling was conducted twice over a year, during the wet seasons (March-May) and (Oct-Dec) of 2012–2013, on unprotected public land. No protected species were sampled.

**Fig 1:**
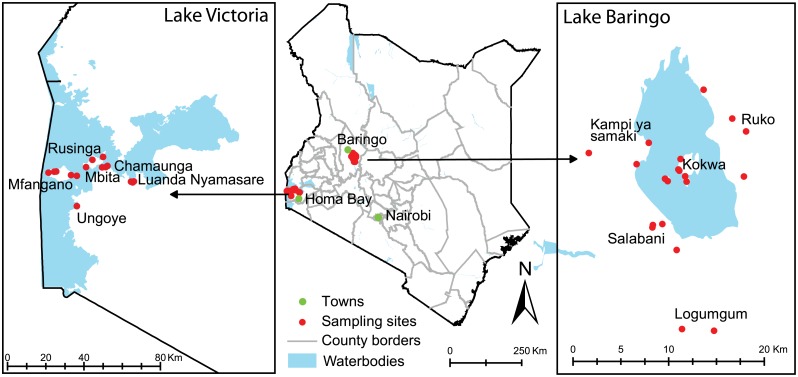
Map of mosquito sampling areas near Lakes Victoria and Baringo in Kenya.

In Homa Bay County, a total of five carbon dioxide baited CDC light traps and two BG sentinel traps were set over two trap nights per season at each of six sampling areas on the mainland shores of Lake Victoria in Ungoye, Luanda Nyamasare and Mbita (0°26’06.19” S, 34°12’53.13”E; elevation ~1,137 m) and the adjacent islands of Mfangano, Rusinga, and Chamaunga (Fig 1). The region is characterized by an equatorial tropical climate with an average minimum temperature of 16°C and an average maximum temperature of 28°C. The area experiences two rainy seasons: the long rainy season between March and June and the short rainy season between October and December. The average annual rainfall during the sampling period 2011–2013 was 1,536 mm (*icipe*-TOC meteorological station).

In Baringo County, a total of 21 CDC light traps and two BG sentinel traps were deployed over two trap nights per season in the five sampling areas along the shores of Lake Baringo in Kampi ya Samaki, Salabani, and Ruko Conservancy and Logumgum (a remote area with an oxbow lake filled with thick swampy marsh that is slightly distant from Lake Baringo but was a hotspot for 2006–2007 RVF outbreak) [9], as well as the Island of Kokwa (Fig 1). Baringo County is located in the Rift Valley Province of Kenya, 250 km northwest of Nairobi. The area is semi-arid and consists of Acacia-Commiphora bushlands and impenetrable thickets of *Prosopies juliflora* that are displacing native trees and reducing grazing areas. Harsh physical and climatic conditions have led to a sparsely populated district where the local communities rely on pastoralism and on limited crop production. Lake Baringo (0°36'56.4"N 36°03'36.8"E, elevation ~980 m) has an annual rainfall range of 300 to 700 mm, and the daily temperature varies between 16°C and 42°C.

### Mosquito trapping

Mosquitoes were trapped outdoors, proximal to human habitation, in villages with CO_2_-baited CDC light traps placed at sunset and collected between 6:00am and 9:00am the following morning and BG sentinel traps with BG-Lure (Human skin scented) odor baits in the evenings between 5:00pm and 6:00pm. After collection, mosquitoes were anesthetized using triethylamine [11] and identified on a chilled surface (paper towels over -80°C icepacks) to species level based on their morphological features [12]. Blood-fed mosquitoes were individually separated in vials and cryopreserved in liquid nitrogen for transportation to the laboratory where they were stored for bloodmeal analysis in -80° freezers.

### Nucleic acid extraction

DNA was extracted from frozen intact blood-fed mosquitoes using two extraction protocols, DNeasy Blood and Tissue Kit (QIAGEN, Valencia, CA) extraction protocol was used initially, and the MagNA 96 Pure DNA and Viral NA Small Volume Kit (Roche Applied Science, Penzberg, Germany) was subsequently used in an automated MagNA Pure 96 extraction system (Roche Applied Science) to increase throughput. The first batch of mosquitoes (from Homa Bay County) was extracted using DNeasy Blood and Tissue Kit (QIAGEN, Valencia, CA) extraction protocol. The 258 engorged abdomens were separated from the rest of the body using sterile dissection pins and homogenized in phosphate buffered saline (PBS). Blood was extracted following the manufacturer’s instructions. In the second batch of 214 samples from Baringo County, we used the MagNA Pure 96 DNA and Viral NA Small Volume Kit (Roche Applied Science, Penzberg, Germany). Individual blood-fed mosquitoes were homogenized whole for 5 sec in 0.5 ml screw-cap tubes (Sarstedt, Newton, NC) filled with 750 mg of 2.0 mm, 150 mg of 0.1 mm zirconia/yttria stabilized zirconium oxide beads (Glen Mills, Clifton, NJ), and 350 μl of PBS before DNA extraction in an automated MagNA Pure 96 extraction system (Roche Molecular Systems, Pleasanton, CA).

### Primer design

Although *cyt b* primers have already been used successfully for HRM-based identification of Chagas disease vectors (Triatominae bugs; *Triatoma sp*. and *Rhodnius sp*.) [7], our preliminary investigations found that these amplicons could not reliably differentiate some of the diversity of potential mosquito bloodmeal host species in East Africa. To improve the resolution of specific bloodmeal host identifications, we designed primers for PCR-HRM analysis to compliment HRM analyses based on *cyt b* primers [7,13]. We generated a multiple alignment of complete vertebrate mitochondrial genomes (Table 1), identified regions of high and low conservation across broad taxa and manually designed primers in conserved regions that flank highly polymorphic sequences of no more than 300 base pairs (bp). We then aligned these primer pair sequences with multiple alignments of invertebrate mitochondrial genomes (GenBank Accessions: AY140887, HM132112, EU352212, NC_006817, NC_014574, NC_015079, NC_014275, HQ724614, DQ146364, MSQMTCG) and identified primer pairs that could discriminately amplify vertebrate sequences in an arthropod vector background. We empirically identified a set of primers that amplify a 190–250 bp fragment, depending on the vertebrate species, of the 16S ribosomal (r) RNA gene that generates distinct HRM profiles for a broad range of bloodmeal hosts.

**Table 1:** Vertebrate mitochondrial genomes aligned for primer design.

Vertebrate Class	Common name	Species	GenBank Accession
Mammalia	Human	*Homo sapiens*	KC569547
	Cow	*Bos taurus*	KC153975
	Goat	*Capra hircus*	NC_005044
	Dog	*Canis lupus familiaris*	NC_002008
	Horse	*Equus caballus*	NC_001640
	Zebra	*Equus zebra hartmannae*	JX312719
	Greyvi zebra	*Equus grevyi*	JX312725
	Baboon	*Papio cynocephalus*	JX946199
	Mouse	*Mus musculus*	JX945979
	Cane rat	*Thryonomys swinderanius*	NC_002658
	Hyrax	*Dendrohyrax dorsalis*	NC_010301
	Elephant	*Loxodonta africana*	NC_000934
	Hippopotamus	*Hippopotamus amphibius*	NC000889
Aves	Chicken	*Gallus gallus*	AY235570
Amphibia	Asian common toad	*Bufo melanostictus*	NC_005794
	African common toad	*Amietophrynus regularis*	DQ158485
	Seoul frog	*Rana chosenica*	NC_016059
Reptilia	Galapagos tortoise	*Geochelone nigra*	JN999704
	Nile monitor	*Varanus niloticus*	NC_008778

### Molecular bloodmeal identification

For identification of vertebrate hosts in mosquito bloodmeals, we complimented the HRM profile differences observed using the established 383 bp *cyt b* gene amplicon primers with the 200 bp amplicons obtained using our newly designed 16S rRNA gene primers (Table 2). Using mosquito DNA extracts as negative controls, we carried out PCRs in final volumes of 10 μl with 0.5 μM concentrations of each primer, using 5X Hot Firepol Evagreen HRM Mix (Solis BioDyne, Tartu, Estonia) and 1 μl of DNA template. The thermal cycling conditions used for *cyt b* primers were as follows: Initial denaturation was done for 1 min at 95°C, followed by 35 cycles of denaturation at 95°C for 30 sec, annealing at 58°C for 20 sec, and extension at 72°C for 30 sec followed by a final extension at 72°C for 7 min. Cycling conditions for 16S ribosomal DNA fragment were similar to those of *cyt b* except for an annealing temperature of 56°C. All PCR reactions were conducted on an HRM capable Rotor-Gene Q real time PCR thermocycler (QIAGEN, Hannover, Germany).

**Table 2:** Oligonucleotide primers used.

Target	Primer name	Primer sequence	Citation
Vertebrate *cyt b*	Cytb For	5'-CCC CTC AGA ATG ATA TTT GTC CTC A-3'	[7,13]
	Cytb Rev	5'-CAT CCA ACA TCT CAG CAT GAT GAA A-3'	
Vertebrate 16S	Vert 16S For	5'-GAG AAG ACC CTR TGG ARC TT-3'	-
	Vert 16S Rev	5'-CGC TGT TAT CCC TAG GGT A-3'	
*Phlebovirus*	Phebo F1	5'-AGT TTG CTT ATC AAG GGT TTG ATG C-3'	[14]
	Phlebo F2	5'-GAG TTT GCT TAT CAA GGG TTT GAC C-3'	
	Phlebo R	5'-CCG GCA AAG CTG GGG TGC AT-3'	
*Orthobunyavirus*	BUN F	5'-CTG CTA ACA CCA GCA GTA CTT TTG AC-3'	[14]
	BUN R	5'-TGGAGGGTAAGACCATCGTCAGGAACTG-3'	
*Alphavirus*	Vir 2052 F	5'-TGG CGC TAT GAT GAA ATC TGG AAT GTT-3'	[15]
	Vir 2052 R	5'-TAC GAT GTT GTC GTC GCC GAT GAA-3'	
*Flavivirus*	1NS5F	5'-GCA TCT AYA WCA YNA TGG G-3'	[16]
	1NS5R	5'-CCA NAC NYN RTT CCA NAC-3'	
	2NS5F	5'-GCN ATN TGG TWY ATG TGG-3'	
	2NS5R	5'-TRT CTT CNG TNG TCA TCC-3'	

Following PCR, melting-curve analysis of amplicons was conducted by gradually increasing the temperature in 0.1°C, 2 second increments from 65°C to 90°C and recording and plotting changes in fluorescence with changes in temperature (dF/dT). DNA extracted from known vertebrate blood samples were used as standard reference controls and included cow, goat, sheep and human clinical samples from Suba district (within afore-mentioned ethics approval), as well as Swiss mouse, rabbit, and chicken blood samples sourced from *icipe*’s animal rearing unit. DNA from mosquito legs were used as negative controls. PCR-HRM protocols were validated for accuracy and sensitivity using reference controls. Ten microliters of human, mouse, rabbit and chicken reference control blood were serially diluted ten-fold in PBS to 10^−7^. DNA from three 1 μl replicates of each diluent was extracted using DNeasy Blood and Tissue Kit (QIAGEN, Valencia, CA) extraction protocol. HRM analysis was carried out using the Rotor-Gene Q software v2.1 with normalization regions between 76.0–78.0°C and 89.50–90.0°C. Bloodmeal sources were identified by comparison of melting profiles with those of the reference control species. Representative samples that generated unknown HRM curves either from the *cyt b* or 16S PCR amplicons, that did not match our standard reference controls were purified with ExoSAP-IT (USB Corporation, Cleveland, OH) to remove unincorporated dNTPs and PCR primers before submission for amplicon sequencing at Macrogen (Seoul, South Korea). Returned sequences were edited in Geneious 7.0.5 and queried in GenBank nr using the Basic Local Alignment Search Tool (http://blast.ncbi.nlm.nih.gov/Blast.cgi), and aligned using Geneious Software to homologous sequences. For identification of specific vertebrate host species, we used a homology cutoff of 97%-100% identity with a GenBank *e*-value threshold of 1.0*e*-130 for the *cyt b* sequences and 1*e*-75 for the 16S sequences. Based on multiple alignments, particularly among the bird samples, some identifications were limited to higher taxonomic classifications.

### Identification of arbovirus infections

Identification of arbovirus infections was performed on 214 mosquito samples comprising the second batch from Baringo. Fifty microlitre aliquots of homogenates were inoculated on 24-well culture plates (Nunc Culture Treated Multidishes, Thermo Scientific, USA) with confluent monolayers of Vero cell line grown in growth media (minimum essential medium with 10% fetal bovine serum (FBS), 2% glutamine, 100 U/ml penicillin, 100 μg/ml streptomycin, and 1μl/ml amphotericin B) and observed for virus induced cytopathology for 14 days. Culture wells that showed cytopathic effects were harvested and RNA extracted using the MagNA 96 Pure DNA and Viral NA Small Volume Kit (Roche Applied Science) in a MagNA Pure 96 (Roche Applied Science) automated extractor, followed by reverse transcription using the High Capacity cDNA Reverse Transcription Kit (Life technologies, Carlsbad, CA). Using published primers (Table 2), we screened for phleboviruses [14], orthobunyaviruses [14] and alphaviruses [15] by PCR, as well as flaviviruses by nested PCR [16] in 10 μl reactions using 1μl cDNA template, 5 μl MyTaq HS master mix (Bioline Reagents Limited, London, UK) and 1 μl of 50 μM SYTO-9 saturating intercalating dye (Life technologies, Carlsbad, California). Laboratory arboviral stocks of diverse Kenyan isolates [17] were used as standard reference controls. Viral amplicons were initially identified by HRM analysis and then confirmed by sequencing at Macrogen (Seoul, South Korea) after amplicon purification with ExoSAP-IT (USB Corporation, Cleveland, OH).

## Results

We sampled a total of 58,497 mosquitoes from both study areas. More mosquitoes were collected in Baringo County (68.31%) than in Homa Bay County (31.69%). Out of the total collection, 472 (0.81%) mosquitoes were blood-fed (258 from Homa Bay County, shores of Lake Victoria; 214 from Baringo County) and analyzed. In Homa Bay, blood-fed mosquito samples were composed of 15 species in all the six sampling sites with the highest proportion from the Mfangano Island (27.13%), while Rusinga Island had the least (5.81%). *Culex naivei* (9.3%), and *Culex pipiens* (5.42%) were the predominant blood-fed species in Mfangano, while *Aedes mettalicus* (0.38%) was the least abundant (Table 3). In Baringo County, the samples constituted 11 mosquito species in all five sampling areas with the highest frequency of blood-fed mosquitoes in Logumgum (37.85%). On average, blood-fed *Mansonia africana* was abundant in all sampling areas, while *Ae*. *aegypti* (0.93%) sampled at Salabani and *Ae*. *vittatus* (0.93%) sampled from Ruko wildlife conservancy were the least abundant blood-fed species from Baringo. The percentage abundance of blood-fed mosquitoes sampled in both study areas are represented in Table 3.

**Table 3:** Numbers of blood-fed mosquito species captured on sampling sites of Homa Bay and Baringo Counties of Kenya.

	Homa Bay County							Baringo County					
	Mainland sites			Island sites				Mainland sites					
Species	Mbita	Ungoye	Luanda Nyamasare	Mfangano	Chamaunga	Rusinga	Homa Bay Total	Kampi ya samaki	Ruko	Salabani	Logumgum	Kokwa Island	Baringo Total
*Ad*. *africana*											17(7.94%)		17 (7.94%)
*Ae*. *aegypti*				4 (1.55%)		1 (0.38%)	5 (1.94%)			2(0.93%)			2 (0.93%)
*Ae*. *hirsutus*				3 (1.16%)			3 (1.16%)						
*Ae*. *mettalicus*				1 (0.38%)	6 (2.32%)		7 (2.71%)						
*Ae*. *vittatus*								1 (0.46%)	2 (0.93%)				3 (1.40%)
*An*. *coustani*			4 (1.55%)	10 (3.87%)	8 (3.1%)	4 (1.55%)	26 (10.08%)	3 (1.4%)	11 (5.14%)		15 (7.01%)	3 (1.4%)	32 (14.95%)
*An*. *funestus*					2 (0.77%)		2 (0.78%)	1 (0.46%)					1 (0.47%)
*An*. *gambiae*	5 (2.33%)		7 (2.71%)	7 (2.71%)		1 (0.38%)	20 (7.75%)	2 (0.93%)	2 (0.93%)		4 (1.87%)		8 (3.74%)
*An*. *pharoensis*	2 (0.77%)						2 (0.78%)						
*Cx*. *naivei*				24 (9.3%)			24 (9.30%)						
*Cx*. *pipiens*	14 (5.42%)	6 (2.32%)	14 (5.42%)	14 (5.42%)		3 (1.16%)	51 (19.77%)	3 (1.4%)		4 (1.87%)		7 (3.27%)	14 (6.54%)
*Cx*. *poicilipes*					14 (5.42%)		14 (5.43%)		4 (1.87%)				4 (1.87%)
*Cx*. *univitatus*	9 (3.48%)	1 (0.38%)	7 (2.71%)		6 (2.32%)		23 (8.91%)	1 (0.46%)		9 (4.2%)	5 (2.33%)		15 (7.01%)
*Cx*. *vansomerini*	5 (2.33%)						5 (1.94%)						
*Ma*. *africana*	14 (5.42%)	3 (1.16%)	11 (4.26%)	7 (2.71%)		1 (0.38%)	36 (13.95%)	15 (7.01%)	15 (7.01%)	13 (6.07%)	32 (14.95%)	5 (2.33%)	80 (37.38%)
*Ma*. *uniformis*	9 (3.48%)		13 (5.03%)		2 (0.77%)	5 (2.33%)	29 (11.24%)	9 (4.2%)	3 (1.4%)	6 (2.8%)	8 (3.74%)	12 (5.6%)	38 (17.76%)
*Mm*. *splendens*	11 (4.26%)						11 (4.26%)						
**Totals**	**69 (26.74%)**	**10 (3.87%)**	**56 (21.7%)**	**70 (27.13%)**	**38 (14.72%)**	**15 (5.81%)**	**258**	**35 (16.35%)**	**37 (17.29%)**	**34 (15.88%)**	**81 (37.85%)**	**27 (12.61%)**	**214**

Bloodmeal sources were identified from 445 mosquitoes, representing 94.27% of captured blood-fed mosquitoes. In Homa Bay, bloodmeal sources of mosquitoes **(** Table 4) ranged from humans (15.89%) to domestic (reared by humans) (53.48%) and peridomestic (common within homesteads) (11.24%) animals, as well as diverse species of wild birds (13.56%). Bloodmeal sources from domestic animals included goats (15.89%; *Capra hircus*), cows (15.50%; *Bos taurus*), sheep (6.98%; *Ovis aries*), dogs (5.43%; *Canis lupus*), chicken (5.43%; *Gallus gallus*) and donkeys (4.26%; *Equus asinus*), while peridomestic bloodmeal sources included rodents (2.33%; *Arvicanthis niloticus*, *Rattus norvegicus* and *Mus musculus*), frogs (6.20%; *Ptychadena nilotica* and *Ptychadena anchietae*), toads (1.94% *Bufo regularis*) and two bat species (*Rhinolophus ferrumequinum* and *Micropteropus pusillus*). Wildlife included cane rats (2.33%; *Thryonomys swinderianus*) and wild birds (11.24%) comprising muscovy ducks (*Cairina moschata*), crows (Family: Corvidae), grey heron (*Ardea sp*.), doves (Family: Columbidae), two weaver bird species (Family: Ploceidae), blue-naped mousebirds (*Urocolius macrourus*), cattle egrets (*Bubulcus ibis*) and two passerine bird species (Order: Passeriformes) (Table 5). Similarly, in Baringo, bloodmeal sources of mosquitoes (Table 6) ranged from human (11.21%), to domestic (63.08%) and peridomestic animals (6.07%), but included pigs (*Sus scrofa*) and more wildlife species (12.15%) like baboons (*Papio sp*.), hippopotamus (*Hippopotamus amphibius*) and crested porcupine (*Hystrix cristata*), black bird (Family: Icteridae), Pheasant (Subfamily: Phasianinae) and rabbit (*Orycytolagus sp*.). Sequence identity e-values with specific Blast matches to GenBank sequences, as well as GenBank accessions of bloodmeal *cyt b* sequences are indicated in Table 7. The alignments of the shorter 16S sequences with their closest Blast hits are shown in S1 Fig.

**Table 4:** Number of bloodmeal sources of mosquito species sampled in Homa Bay County.

Sampling area	Species	N	Human	Chicken	Cow	Dog	Donkey	Goat	Sheep	Cane rat	Toad	Rodent	Birds	Wild Duck	Frog	Bat	ND
Mbita	*Cx*. *pipiens* ^#^	14	5	0	1	2	0	5	0	0	0	0	2	0	0	0	0
	*Ma*. *africana*	14	2	0	6	1	1	3	1	0	0	0	0	0	0	0	0
	*Ma*. *uniformis*	9	0	1	2	0	1	3	1	0	0	0	0	1	0	0	0
	*Cx*. *univittatus*	9	2	0	0	1	2	0	1	0	1	0	2	0	0	0	0
	*An*. *gambiae*	5	3	0	0	1	0	0	0	0	0	1	0	0	0	0	0
	*Mm*. *splendens*	11	0	0	0	0	0	0	0	0	0	0	2	0	9	0	0
	*Cx*. *vansomerini*	5	1	1	1	0	0	1	0	0	0	0	1	0	0	0	0
Luanda Nyamasare	*An*. *coustani*	4	0	0	2	0	0	0	1	0	0	0	1	0	0	0	0
	*An*. *gambiae*	7	5	0	1	0	0	1	0	0	0	0	0	0	0	0	0
	*Cx*. *pipiens*	14	4	2	3	0	0	3	1	0	0	0	1	0	0	0	0
	*Cx*. *univittatus*	7	2	0	1	0	1	0	0	0	0	0	3	0	0	0	0
	*Ma*. *africana*	11	0	0	6	0	2	3	0	0	0	0	0	0	0	0	0
	*Ma*. *uniformis*	13	1	0	4	0	0	4	4	0	0	0	0	0	0	0	0
Ugoye	*Cx*. *pipiens*	6	2	0	1	0	0	2	0	0	0	0	0	0	0	0	1
	*Cx*. *univittatus*	1	0	0	0	0	0	1	0	0	0	0	0	0	0	0	0
	*Ma*. *africana*	3	0	0	1	0	0	2	0	0	0	0	0	0	0	0	0
	*An*. *pharoensis*	2	1	0	0	0	0	0	1	0	0	0	0	0	0	0	0
Mfangano	*Cx*. *naivei*	24	6	2	2	3	0	2	1	1	0	1	4	0	0	0	2
	*Cx*. *pipiens*	14	4	2	0	0	1	3	1	0	0	0	0	0	1	1	1
	*An*. *coustani*	10	0	1	3	1	1	2	2	0	0	0	0	0	0	0	0
	*An*. *gambiae*	7	1	0	0	1	0	1	1	2	1	0	0	0	0	0	0
	*Ma*. *africana*	7	2	1	1	1	0	0	1	0	1	0	0	0	0	0	0
	*Ae*. *aegypti*	4	0	0	1	1	0	0	1	0	0	1	0	0	0	0	0
	*Ae*. *hirsutus*	3	0	2	0	0	0	0	0	0	0	0	0	0	1	0	0
	*Ae*. *mettalicus*	1	0	1	0	0	0	0	0	0	0	0	0	0	0	0	0
Chamaunga	*Cx*. *poicilipes*	14	0	0	0	0	1	1	0	3	1	2	5	0	0	1	0
	*An*. *coustani*	8	0	0	0	0	1	1	0	0	0	1	1	0	1	0	3
	*Cx*. *univittatus*	6	0	0	1	0	0	1	0	0	0	0	2	1	0	0	1
	*Ae*. *metalicus*	6	0	0	1	1	0	0	0	0	1	0	1	1	1	0	0
	*An*. *funestus*	2	0	0	0	0	0	0	0	0	0	0	1	0	1	0	0
	*Ma*. *uniformis*	2	0	0	0	0	0	0	0	0	0	0	1	1	0	0	0
Rusinga	*An*. *coustani*	4	0	0	0	0	0	0	0	0	0	0	0	2	1	0	1
	*Ma*. *uniformis*	5	0	1	1	0	0	1	0	0	0	0	1	0	1	0	0
	*Ma*. *africana*	1	0	0	1	0	0	0	0	0	0	0	0	0	0	0	0
	*Cx*. *pipiens*	3	0	0	0	0	0	1	1	0	0	0	1	0	0	0	0
	*An*. *gambiae*	1	0	0	0	0	0	1	0	0	0	0	0	0	0	0	0
	*Ae*. *aegypti*	1	0	0	0	1	0	0	0	0	0	0	0	0	0	0	0
**Total**		#	**41 (15.89%)**	**14 (5.42%)**	**40 (15.50%)**	**14 (5.43%)**	**11 (4.26%)**	**42 (16.28%)**	**18 (6.98%)**	**6 (2.33%)**	**5 (1.94%)**	**6 (2.33%)**	**29 (11.24%)**	**6 (2.33%)**	**16 (6.20%)**	**2 (0.78%)**	**9 (3.49%)**

N = Number of mosquitoes analyzed, ND = Not determined.

^#^mixed blood meal cases.

**Table 5:** Bird species represented in mosquito bloodmeals.

Birds represented in mosquito bloodmeal	Mosquito species	Study site	GenBank Accession (% identity; *e*-value)
Weaver bird (*Ploceus baglaflecht reichnowi*)	*Cx*. *naivei* (2)	Mfangano	AY283898 (97% 16S; 9*e*-76)
	*An*. *gambiae* (1)	Logumgum	
	*Ae*. *metalicus* (1)	Chamaunga	
	*An*. *funestus* (1)	Chamaunga	
Weaver bird (Family: Ploceidae)	*Cx*. *pipiens*(1)	Luanda Nyamasare	AY283898 (96% 16S; 9*e*-76)
	*Cx*. *pipiens* (1)	Kokwa	
	*An*. *coustani* (3)	Kokwa	
	*Ma*. *uniformis* (1)	Logumgum	
Blackbird (Family: Icteridae)	*Ma*. *uniformis* (1)	Logumgum	JX516070 (94% 16S; 4*e*-54)
	*Ma*. *africana* (1)	Logumgum	
Swallow (*Hirundo rustica*)	*Mm*. splendens (2)	Mbita	AB042382 (99% 16S; 7*e*-82)
Blue-naped mousebird (*Urocolius macrourus*)	*Cx*. *vansomereni* (1)	Mbita	AF173589 (99% 16S; 3*e*-90)
	*Cx*. *pipiens*(1)	Mbita	
	*Cx*. *univittatus* (3)	Luanda Nyamasare, Chamaunga	
	*Ma*. *uniformis* (1)	Rusinga	
Cattle egret (*Bubulcus ibis*)	*Cx*. *poicilipes* (2)	Chamaunga	KJ190945 (99% 16S; 1*e*-890)
	*Ad*. *africana* (1)	Logumgum	
Chicken (*Gallus gallus*)	*Ma*. *uniformis* (1)	Mbita	AY236430 (100% 16S; 2*e*-82)
	*Cx*. *vansomereni* (1)	Mbita	
	*Cx*. *pipiens*(8)	Luanda Nyamasare, Mfangano, Kampi ya Samaki, Kokwa	
	*Cx*. *naivei* (2)	Mfangano	
	*An*. *coustani* (6)	Mfangano, Kokwa, Logumgum	
	*Ma*. *africana* (8)	Mfangano, Kampi ya Samaki, Salabani, Logumgum	
	*Ae*. *hirsutus* (2)	Mfangano	
	*Ae*. *metalicus* (1)	Mfangano	
	*Ma*. *uniformis* (6)	Rusinga, Kampi ya Samaki, Salabani, Mbita	
	*An*. *gambiae* (1)	Kampi ya Samaki	
	*Ae*. *aegypti* (1)	Salabani	
	*Cx*. *univittatus* (1)	Logumgum	
	*Cx*. *poicilipes*(1)	Ruko	
Pheasant (Subfamily: Phasianinae)	*Ma*. *uniformis* (3)	Kokwa	EU165707 (92% 16S; 2*e*-51)
Grey heron (*Ardea cinerea*)	*Ma*. *uniformis*	Salabani	KJ190947 (100% 16S; 2*e*-77)
	*Cx*. *poicilipes* (3)	Salabani	
Muscovy duck (*Cairina moschata*)	*Cx*. *univittatus* (1)	Kampi ya Samaki	EU755254 (100% 16S; 2*e*-81)
	*Ma*. *uniformis* (2)	Mbita, Chamaunga	
	*An*. *coustani* (2)	Rusinga	
Passerine bird (Order: Passeriformes)	*Cx*. *naivei* (1)	Mfangano	KM078809 (96%; 2*e*-71)
	*Cx*. *coustani* (1)	Chamaunga	
	Cx. univittatus(1)	Logumgum	
	Cx. univittatus (1)	Logumgum	
Passerine bird (Order: Passeriformes)	*Cx*. *naivei* (1)	Mfangano	FJ465224 (94%; 6*e*-68)
	*Cx*. *pipiens* (1)	Mbita	
	*Ma*. *uniformis* (1)	Chamaunga	
Dove (Family: Columbidae)	*Cx*. *univittatus* (3)	Ruko, Mbita	KC984248 (96% 16S; 5*e*-86)
	*Ma*. *africana* (1)	Ruko	
	*An*. *coustani* (2)	Ruko	
Crow (Family: Corvidae)	*Cx*. *univittatus* (1)	Salabani	AF171067 (91% *cyt b*, 1*e*-112)

**Table 6:** Number of bloodmeal sources of mosquito species sampled in Baringo County.

Sampling area	Species	N	Human	Chicken	Cow	Dog	Donkey	Goat	Sheep	Pig	Frog	Hippo	Baboon	Bird	Rabbit	Porcupine	Rodent	ND
Kampi ya samaki	*Ma*. *africana*	15	2	1	6	0	2	2	2	0	0	0	0	0	0	0	0	0
	*Ma*. *uniformis*	9	0	1	2	0	0	3	2	0	0	0	0	0	0	0	1	0
	*Cx*. *pipiens*	3	1	1	0	0	0	0	0	0	0	0	0	0	0	1	0	0
	*An*. *coustani*	3	1	1	0	0	0	1	0	0	0	0	0	0	0	0	0	0
	*An*. *gambiae*	2	1	1	0	0	0	0	0	0	0	0	0	0	0	0	0	0
	*Cx*. *univittatus*	1	0	0	0	1	0	0	0	0	0	0	0	0	0	0	0	0
	*Ae*. *vittatus*	1	0	0	0	0	0	0	1	0	0	0	0	0	0	0	0	0
	*An*. *funestus*	1	0	1	0	0	0	0	0	0	0	0	0	0	0	0	0	0
Ruko	*Ma*. *africana*	15	1	1	6	1	1	2	0	0	1	0	1	1	0	0	0	1
	*An*. *coustani*	11	1	1	2	0	1	1	0	1	2	0	0	2	0	0	0	0
	*Cx*. *poicilipes*	4	0	1	0	0	0	1	0	0	1	0	0	0	0	0	0	1
	*Ma*. *uniformis*	3	1	1	0	0	0	0	0	0	0	0	0	0	0	0	0	1
	*An*. *gambiae*	2	1	0	0	0	0	1	0	0	0	0	0	0	0	0	0	0
	*Ae*. *vittatus*	2	1	1	0	0	0	0	0	0	0	0	0	0	0	0	0	0
Salabani	*Ma*. *africana*	13	1	3	2	0	2	3	1	0	1	0	0	0	0	0	0	0
	*Cx*. *univittatus*	9	1	0	1	0	2	1	0	0	0	0	1	1	0	0	1	1
	*Cx*. *pipiens*	4	3	0	0	0	0	0	1	0	0	0	0	0	0	0	0	0
	*Ma*. *uniformis*	6	0	2	0	1	0	0	1	0	0	0	0	1	0	0	0	1
	*Ae*. *aegypti*	2	0	1	1	0	0	0	0	0	0	0	0	0	0	0	0	0
Kokwa	*Ma*. *uniformis*	12	1	0	2	0	0	1	2	0	0	1	0	3	0	0	0	2
	*Cx*. *pipiens* *	7	1	1	0	0	0	0	0	0	2	0	0	2	0	0	0	1
	*Ma*. *africana*	5	0	0	1	0	1	1	1	0	1	0	0	0	0	0	0	0
	*An coustani*	3	0	1	0	0	0	0	1	0	0	0	0	1	0	0	0	0
Logumgum	*Ma*. *africana* * ^#^	32	3	2	1	1	3	9	4	1	1	1	1	1	0	1	2	3
	*Ad*. *africana* *	17	2	0	3	1	0	1	4	1	1	0	0	1	1	0	0	2
	*An*. *coustani* *	15	1	1	1	1	2	0	3	2	0	1	0	1	0	0	0	2
	*Ma*. *uniformis*	8	0	1	1	0	0	1	2	0	0	0	0	1	0	0	0	2
	*Cx*. *univittatus*	5	1	1	0	1	0	0	0	0	0	0	0	1	0	0	0	1
	*An*. *gambiae*	4	0	0	1	1	0	1	0	0	0	0	0	1	0	0	0	0
**Total**		**214**	**24 (11.21%)**	**25 (11.21%)**	**30 (14.02%)**	**8 (3.74%)**	**14 (6.54%)**	**29 (13.55%)**	**25 (11.68%)**	**5 (2.34%)**	**10 (4.67%)**	**3 (1.40%)**	**3 (1.40%)**	**17 (7.94%)**	**1 (0.47%)**	**2 (0.93%)**	**3 (1.40%)**	**18 (8.41%)**

N = Number of mosquitoes analyzed, ND = Not determined.

^#^mixed blood meal cases.

*virus positive cases.

**Table 7:** Sequence identity and e-values of all mosquito bloodmeal sources with specific BLAST matches to GenBank sequences (Accessed May 25, 2015).

Blood meal sources	Study *cyt b* sequence	*cyt b* (% identity; *e*-value)	16S (% identity; *e*-value)
Human (*Homo sapiens*)	KP858485	KJ801974 (100%; 1*e*-150)	KP218948 (99%; 4*e*-79)
Goat (*Capra hircus*)	KP858486	FM205715 (100%; 2*e*-153)	KP195268 (100%; 2*e*-72)
Cattle (*Bos taurus*)	KP858487	EU365345 (100%; 1*e*-151)	KJ789953 (99%; 3*e*-70)
Sheep (*Ovis aries*)			KP998473 (98%; 4*e*-79)
Dog (*Canis familiaris)*	KP858493*	DQ309764* (100%; 5*e*-150)	KF661075 (99%; 3*e*-91)
Donkey (*Equus asinus*)			KM881681 (99%; 3*e*-85)
Pig (*Sus scrofa*)	KP858488	KJ652503 (100%; 9*e*-148)	
Rat (Subfamily: Murinae)			AF141228 (92%; 4*e*-59)
Baboon (*Papio sp*.)	KP858489	JX946199 (100%; 1*e*-146)	
House mouse (*Mus musculus*)	KP858490	KP260517 (100%; 7*e*-144)	
Brown rat (*Rattus norvegicus*)	KP858491	KP241960 (100%; 1*e*-146)	
Cane rat (*Thryonomys sp*.)	KP844654	AJ301644 (97%; 5*e*-140)	
Rabbit (*Oryctolagus sp*.)	KP858492	HG810791 (100%; 4*e*-146)	
Crested porcupine (*Hystrix cristata*)	KP858484	FJ472572 (100%; 7*e*-144)	
Fruit bat (*Micropteropus pusillus*)			JN398183 (100%; 6*e*-72)
Hippopotamus (*Hippopotamus amphibius*)	KP844655	AP003425 (99%; 3*e*-132)	
Weaver bird (*Ploceus baglaflecht reichnowi*)			AY283898 (97%; 9*e*-76)
Weaver bird (Family: Ploceidae)			AY283898 (96%; 9*e*-76)
Blackbird (Family: Icteridae)			JX516070 (94%; 4*e*-54)
Swallow (*Hirundo rustica*)			AB042382 (99%; 7*e*-82)
Blue-naped mousebird (*Urocolius macrourus*)			AF173589 (99%; 3*e*-90)
Cattle egret (*Bubulcus ibis*)			KJ190945 (99%; 1*e*-89)
Chicken (*Gallus gallus*)			AY236430 (100%; 2*e*-82)
Pheasant (Subfamily: Phasianinae)			EU165707 (92%; 2*e*-51)
Grey heron (*Ardea cinerea*)			KJ190947 (100%; 2*e*-77)
Muscovy duck (*Cairina moschata*)			EU755254 (100%; 2*e*-81)
Passerine bird (Order: Passeriformes)			FJ465224 (94%; 6*e*-68)
Passerine bird (Order: Passeriformes)			KM078809 (96%; 2*e*-71)
Dove (Family: Columbidae)			KC984248 (96%; 5*e*-86)
Crow (Family: Corvidae)	KP844653	AF171067 (91%, 1*e*-112)	
African toad (*Bufo regularis*)			AF220889 (100%; 7*e*-92)
Grass frog (*Ptychadena nilotica*)			DQ525928 (100%; 8*e*-86)
Anchieta's ridged frog (*Ptychadena anchietae*)			GQ183598 (100%; 1*e*-84)

*cyt b like pseudogene

**Fig 2:**
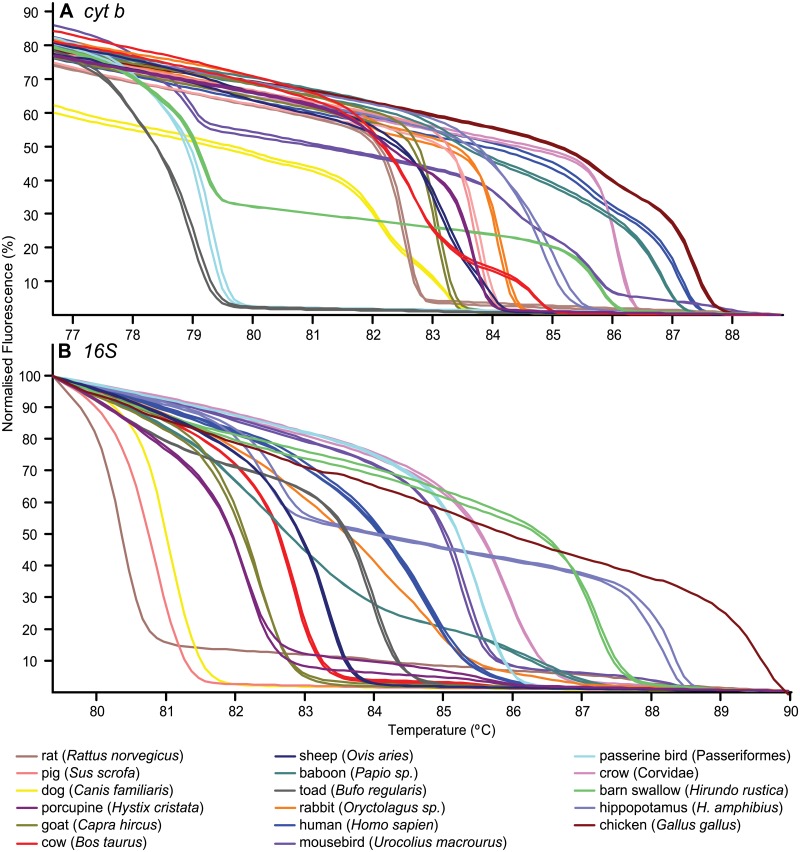
HRM profiles of selected mosquito bloodmeal sources using *cyt b* (A) and 16S rRNA (B). Vertebrate species in the legend are ordered from their lowest to highest 16S rRNA melting temperatures.

**Fig 3:**
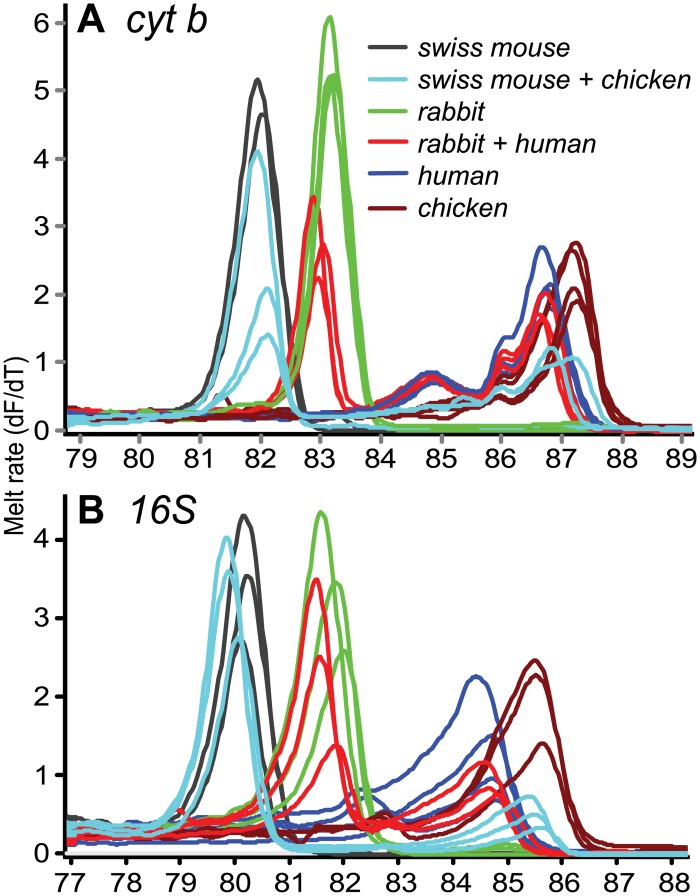
Melt rates of serially diluted pure and mixed blood for calibration of identifications and sensitivity validations using *cyt b* (A) and 16S rRNA (B). Vertebrate species in the legend are ordered from their lowest to highest melting temperatures.

**Fig 4:**
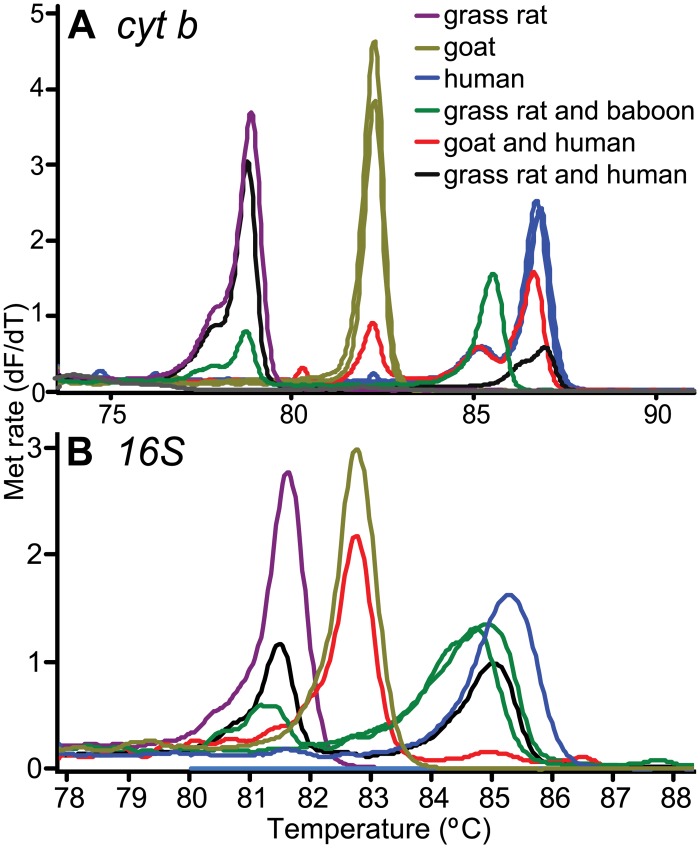
Melt rates of field collected mixed mosquito blood meals using *cyt b* (A) and 16S rRNA (B). Vertebrate species in the legend are ordered from their lowest to highest melting temperatures.

**Fig 5:**
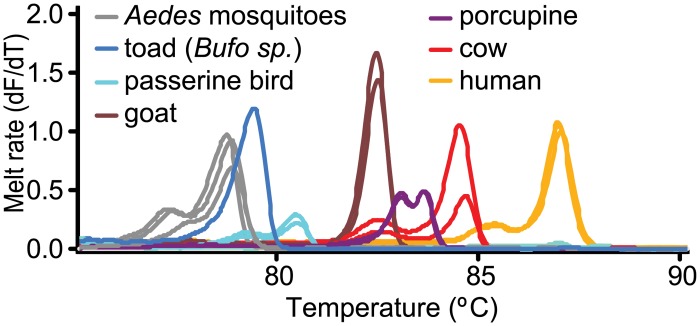
Melt rates of mosquito *cyt b* amplicons alongside selected vertebrate bloodmeal amplicons. Species in the legend are ordered from their lowest to highest melting temperatures.

Distinct HRM profiles of diverse bloodmeal species are shown in Fig 2. Detection of mixed blood meals was evaluated for accuracy and sensitivity from 1 μl triplicate aliquots of ten-fold serial dilutions (up to 10^−7^) of pure and mixed blood. The sensitivity of the two markers in identifying the standard controls is tabulated in Table 8. Detection of mixed and pure blood showed slight variations in melting profiles (Fig 3). Mixed bloodmeals in the field-collected samples were detected in *Cx*. *pipiens* gut from Mbita that had fed on goat and human blood, and two *Ma*. *africana* sampled in Baringo that both had bloodmeals of rat (*Arvicanthis niloticus*) in addition to human or baboon (*Papio sp*.) (Fig 4). Although the *cyt b* marker could more efficiently identify mixed bloodmeals in our field collection than the 16S marker (Fig 4), there were instances where sequencing of *cyt b* amplicons resulted in mosquito-specific sequences (<90% sequence homology) with lower melting temperature ranges than vertebrates (Fig 5). Such was observed in *Ae*. *hirsutus*, *Ae*. *metallicus*, *Ae*. *aegypti*, *Ae*. *vittatus* and *An*. *coustani* that were among the 27 samples in which mosquito DNA amplified instead of their host’s DNA. These were clearly resolved based on 16S HRM analysis, resulting in correct identification of vertebrate host species. We also found that melting profiles of some pairs of vertebrate host species may appear somewhat similar when using products of either *cyt b* or 16S alone, but not across both markers (Fig 2). For instance, pairs of host species such as pig and wild rabbit (*Oryctolagus sp*.) or human and baboon (*Papio sp*.) could be differentiated based on their 16S HRM profiles, but not based on their *cyt b* HRM profiles. Likewise, similar 16S HRM profiles were observed between crested porcupine (*Hystrix cristata*) and goat (*Capra hircus*) and between passerine bird and mousebird bloodmeals that could be differentiated based on their *cyt b* HRM profiles. Therefore, the comparisons of HRM profiles from both loci allowed for more robust species differentiation and identification among many species (Fig 2).

**Table 8:** Dilution factor detection limits of cyt b and 16S rRNA markers of pure and mixed serially diluted blood.

Host	Cytochrome b	16S rRNA
Chicken	10^−4^ (0.1 nl)	10^−4^ (0.1 nl)
Human	10^−6^ (1 pl)	10^−6^ (1 pl)
Rabbit	10^−4^ (0.1 nl)	10^−4^ (0.1 nl)
Swiss mouse	10^−6^ (1 pl)	10^−5^ (10 pl)
Human-Rabbit Mix	10^−4^ (0.1 nl)	10^−5^ (10 pl)
Chicken-Swiss mouse mix	10^−3^ (1 nl)	10^−5^ (10 pl)

Volumes of blood extracted at different dilutions are indicated in brackets.

**Fig 6:**
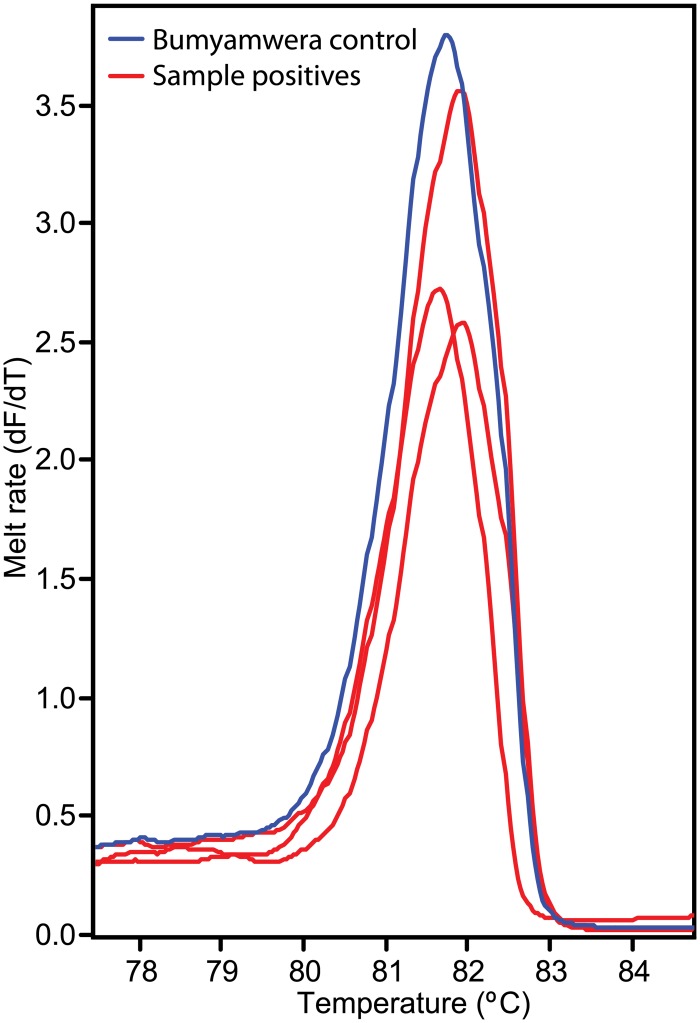
Melt rate profiles of Bunyamwera S segment amplicons detected in blood-fed mosquitoes.

We identified seven out of 214 (3.27%) cell culture wells inoculated with blood-fed mosquito homogenates from Baringo that were suspected to be positive for virus induced cytopathology. Three isolates were successfully amplified and resolved as Bunyamwera Virus by HRM (Fig 6) and confirmed by amplicon sequencing of 199bp non structural gene fragment (98% identity to GenBank accession KM507344; *e*-value 8*e*-92). These Bunyamwera Virus positives included *Aedeomyia africana* that had fed on cattle, *An*. *coustani* that had fed on sheep and *Ma*. *africana* that had fed on man all from Logumgum. From a *Cx*. *pipiens* from Kokwa Island that had fed on human, we also sequenced a 91bp fragment of Sindbis virus (95% identity to GenBank accession KF737350; *e*-value 1e-30). The rest were not fully characterized, as the samples did not amplify.

## Discussion

Blood-fed mosquitoes representing diverse vectors of malaria, arboviruses and filarial worms were sampled in both Baringo and Homa Bay counties. We identified 33 distinct vertebrate hosts of mosquito bloodmeals based on their HRM profiles (Fig 2) across both *cyt b* and 16S gene PCR products. Bloodmeal profiles of vertebrates were specifically matched to sequenced positive controls and novel profiles were identified by sequence analysis. Previously, the *cyt b* marker had been used to resolve 14 vertebrate species as sources of triatomine bug bloodmeals in South America. However, this approach alone could not reliably differentiate the diversity of potential mosquito bloodmeal host species in East Africa. Compared to less mobile triatomine bugs, mosquitoes can fly long distances [18] seeking bloodmeals and some species feed on a range of hosts.

Melting profiles of some pairs of vertebrate host species could only be differentiated clearly based on HRM profiles generated by either *cyt b* or 16S amplicons, but not by both markers (Fig 2). Therefore, comparisons of HRM profiles from both loci allow for more robust species differentiation and identification. Additionally, we were able to identify hosts of mixed bloodmeal sources of individual mosquitoes based on melting profiles with double peaks (Fig 4). While sequencing of DNA fragments may also resolve mixed feedings, sequence analysis of double base calls is costly, difficult and time-consuming [19]. Our HRM validation results (Fig 3) show high sensitivity in identifying both pure and mixed vertebrate samples from below 1 nl blood volumes (Table 8). As mosquito bloodmeals range from 1nl to 6 ml [20], HRM analysis can effectively identify multiple blood feeding in mosquitoes and probably other vectors.

Most bloodmeals identified were from humans and domestic animals, but we also identified mosquitoes that had fed on wildlife hosts in both study areas. More wildlife bloodmeal sources were identified in Baringo County, which has a higher density of wildlife that interfaces with livestock compared to Homa Bay County. As our traps were positioned proximal to human habitation and farms, bloodmeal host proportions are probably biased by increased host diversity at peridomestic and domestic sampling sites.

In Homa Bay County, blood-fed malarial vectors *An*. *gambiae* (7.75%) and *An*. *funestus* (0.78%) were less commonly collected in comparison to other blood-fed species such as *Cx*. *pipiens* (19.77%), *Ma*. *africana* (13.95%), *Ma*. *unformis* (11.24%) and *An*. *coustani* (10.08%) (Table 3). This could be attributed in part to massive malaria eradication programs, as many studies are being conducted in the area that are focused on development of efficient tools for eliminating malaria and malaria vectors [21–25]. Because we set traps outdoors, we mainly trapped *An*. *arabiensis* (78%) among the small number of *An*. *gambiae s*.*l*. mosquitoes. This was probably due to their high abundance and exophilic tendencies compared to *An*. *gambiae s*.*s*, which are documented to be more endophilic in their feeding behavior [26,27]. This finding concurred with previous entomological survey that showed abundance of *An*. *arabiensis* over *An*. *gambiae s*.*s* and *An funestus s*.*l* around Lake Victoria and adjacent habitats [28].

Bloodmeals of *An*. *gambiae s*.*l*, and *Cx*. *pipiens* were identified from the same range of host species, including birds, with the highest proportions being from humans (Tables 4 and 6). This contrasted with previous observations that *Cx*. *pipiens* preferentially feed on birds than on humans [29–31]. Even during the onset of West Nile cases in humans in suburban Chicago, Illinois, the predominant bloodmeals of its principal vector, *Cx*. *pipiens*, were birds [32]. Considering the prominent abundance of blood-fed *Cx*. *pipiens* (19.77%) in Homa Bay County (Table 3) and its substantial human feeding pattern (Table 3), it is surprising that the diverse bulk of wild birds that thrive from fish and aquatic organisms along the shores of Lakes Victoria and Baringo [33] did not form a higher proportion of *Cx*. *pipiens* bloodmeals. Host feeding of *Cx*. *pipiens* in our study areas are dynamic and dependent on the composition and proportion of available host species present. Alternatively, given that sampling was conducted in close proximity to human habitation, it could be interesting to find out if avian hosts form the bulk of *Cx*. *pipiens* diets in wild caught species that are not close to human habitation.

Blood-fed *An*. *coustani* were well represented in the sampling localities of Homa Bay and Baringo Counties (Table 3). Although this species has been implicated in malaria transmission in some countries [34,35], we only found three (Table 6) out of 32 (9.38%) (Table 3) blood-fed *An*. *coustani* mosquitoes with human bloodmeals in Baringo, while none (Table 4) of the 26 (Table 3) blood-fed *An*. *coustani* sampled from Homa Bay County had human bloodmeals. This finding was surprising considering that in Baringo, cattle herders sleep outdoors to protect their livestock from cattle rustlers and night fishing is popular in Homa Bay County and therefore inhabitants of both areas are likely to be prone to bites of outdoor blood-questing mosquitoes.

*Culex univittatus*, which is considered a competent vector of West Nile virus in Africa was collected within the mainland sampling sites and Chamaunga Island in Homa Bay County and from Kampi ya Samaki, Salabani and Logumgum sites of Baringo County. This species contained bloodmeal sources from humans and domestic animals and interestingly, also from birds such as Muscovy duck (*Cairina moschata*) and *Corvus sp*. that have the potential of long distance migration, which could increase the risk of West Nile virus transmission [36,37]. It is worthwhile noting that West Nile virus was recently isolated in North Eastern Kenya from *Culex sp*. and that in the 2006/2007 Rift Valley fever (RVF) outbreak, RVF virus was isolated from a single pool of *Cx*. *univittatus* collected from Baringo [38,39].

Blood-fed *Ma*. *africana* and *Ma*. *uniformis* were widely encountered in samples analyzed from Mbita and Luanda Nyamasare and were the predominant species in Baringo (Table 3). The abundance of *Ma*. *africana* (37.38%) and *Ma*. *uniformis* (17.76%) in Baringo observed in this study concurred with an entomological survey that was conducted in 2013 [40]. This abundance is noteworthy considering that both vectors have been implicated in RVF virus transmission in Baringo in 2007 and we also detected Bunyamwera virus in *Ma*. *africana* that had fed on human. The two *Mansonia* species exhibited diverse feeding choices with bloodmeal sources including humans and domesticated animals like goats, sheep, donkeys and dogs. Other sources of *Mansonia* bloodmeals included hosts that could be classified as disease reservoirs such as the baboons (*Papio sp*.), rodents (*Ra*. *norvegicus*), amphibians (*Pt*. *nilotica*), crested porcupines (*Hy*. *cristata*) and wild migratory birds (*Ca*. *moschata* and *Ar*. *cinerea*). The diverse choices of blood hosts by these two species could increase the cross-species transmission and potential for Bunyamwera, RVF and possibly other arboviruses and filarial worms in Baringo and Homa Bay. Although our findings indicate that the two species of *Mansonia* prefer to feed on domesticated animals over humans, this may be due to indiscriminate feeding patterns, as domestic animals are more common in areas where mosquitoes were sampled and are located outdoors; always susceptible to nocturnal feeding mosquitoes [41].

We found Sindbis virus in *Cx*. *pipiens* sampled in Kokwa Island that had fed on human and Bunyamwera virus in *Ad*. *africana* that had fed on cattle, *An*. *coustani* that had fed on sheep and *Ma*. *africana* that had fed on man. Although Bunyamwera virus has low pathogenicity in vertebrate hosts [42], it has the propensity to reassort with other closely related orthobunyavirus leading to increased virulence in man [15,43]. As Bunyamwera virus has been isolated from diverse mosquito vectors, ticks and vertebrate hosts in East Africa [17,44,45], it appears to have complex transmission cycles that may facilitate the emergence of reassortant viruses [46] of public health and veterinary concern.

Blood-fed mosquito species composition differed between sampling areas, probably due to adaptation to specific environmental niches or oviposition sites (Table 3). This was observed in *Mimomyia splendens* that were all sampled in close proximity to a wastewater treatment pond in Mbita and *Ad*. *africana* that were sampled in Logumgum, close to an oxbow lake in areas with swampy lagoons. *Mansonia sp*. were also found abundantly in these ecological sites and could migrate to homesteads nearby. Occasionally, bloodmeal sources of these groups of mosquitoes were from amphibians (e.g. frogs and toads) with 9/11 (82%) blood-fed *Mm*. *splendens* mosquitoes having fed on grass frog (*Pt*. *nilotica*) (Table 4). *Ma*. *africana*, *Cx*. *univittatus*, *Ae*. *metalicus* and *An*. *gambiae* and *Cx*. *poicilipes* bloodmeals also included African toad (*Bu*. *regularis*). This kind of feeding could be opportunistic when both amphibians and mosquitoes localize in swampy habitats. Alternatively, such feeding behavior may be restricted to specific mosquito species as some mosquito repellants have been isolated from frog skin [47], and mosquitoes may possibly benefit from antimicrobial peptides found in amphibian skin [48].

This study clearly demonstrates the improved resolution of HRM-based bloodmeal analysis by using two distinct molecular markers, revealing broad opportunistic host feeding patterns among mosquito vectors in arbovirus endemic regions of Kenya. The diverse vertebrate sources of vector bloodmeals and the presence of arbovirus such as Bunyamwera further demonstrate that many of the vectors identified in this study have the potential to transmit Bunyamwera and other pathogens from potential wildlife reservoirs to livestock that may act as amplifying hosts and to humans.

## Supporting Information

S1 Fig Multiple alignment of Vertebrate host 16S sequences from mosquito bloodmeals with their closest GenBank matches. (PDF)
