# Supplementary material for: Unraveling Host-Vector-Arbovirus Interactions by Two-Gene High Resolution Melting Mosquito Bloodmeal Analysis in a Kenyan Wildlife-Livestock Interface
Source: PLoS One. 2015 Jul 31;10(7):e0134375. doi: 10.1371/journal.pone.0134375 (PMC4521840; doi:10.1371/journal.pone.0134375)
Supplement: S1 Fig — (PDF) [file pone.0134375.s001.pdf]

	1 10 20 30 40 50 60
Bufo regularis (African toad) bloodmeal (sample B70)	----------------------ACATTTTAATTTT-----TTTAAG-TAGTCTGACTATA
Bufo regularis (AF220889)	----------------------ACATTTTAATTTT-----TTTAAG-TAGTCTGACTATA
Ptychadena anchietae (Anchieta's ridged frog) bloodmeal (Sample B79)	-----------------------CATGGTAATCTT---GGCTTT---TA-TCTA----TT
Ptychadena anchietae (GQ183598)	-----------------------CATGGTAATCTT---GGCTTT---TA-TCTA----TT
Ptychadena nilotica (Grass frog) bloodmeal (Sample B31)	-----------------------CA--CTAACCTTACAGACTCTAG-AA-TCTACTTGTT
Ptychadena nilotica (DQ525928)	-----------------------CA--CTAACCTTACAGACTCTAG-AA-TCTACTTGTT
Hirundo rustica (Barn swallow) bloodmeal (Sample B21)	---------CA---CCTCGGGCTTACTAACT-CACG-GGTGACTGGCTCGCA--------
Hirundo rustica (AB042382)	---------CA---CCTCGGGCTTACTAACT-CACG-GGTGACTGGCTCGCA--------
Urocolius macrourus (mousebird) bloodmeal (Sample B11,22,B20,B229)	------CCCTC---CACCGGACCTATTAATT--GTA-CACTCCTGGCTA-AA--------
Urocolius macrourus (AF173589)	------CCCTC---CACCGGACCTATTAATT--GTA-CACTCCTGGCTA-AA--------
Bubulcus ibis (Cattle Egret) bloodmeal (sample B180)	------CCCTA---CT--GGATCTACTACC---CTA-AAACGCTGGC-CCGC--------
Bubulcus ibis (KJ190945)	------CCCTA---CT--GGATCTACTACC---CTA-AAACGCTGGC-CCGC--------
Gallus gallus (Chicken) bloodmeal (Sample B19)	------------------GGGTCCACCCACA-CATA-AACCCCTGG-TCGAC--------
Gallus gallus (AY236430)	------CCCCA---CT--GGGTCCACCCACA-CATA-AACCCCTGG-TCGAC--------
Gallus gallus variant (Chicken) bloodmeal (Sample B15)	----------------------CTACCCGCA-CA-A-AGTCATTGG-TCGAT--------
Francolinus pintadeanus (EU165707)	------------------GGATTCACCCATA-CA-A-AGCCCTTGG-TCGAC--------
Streptopelia turtur(Dove) bloodmeal (Sample B5)	----------------A--GGCCCACCACTA--GCA-AGATGCTGGCTC-GC--------
Streptopelia turtur (KC984248)	------CCC-A---CTA--GGCCCACCACTA--GCA-AGATGTTGGCTC-AC--------
Cairina moschata (Muscovy duck) bloodmeal (Sample B6)	--------------CT--GGGGCCACTACTAATCGC-AAGGCATGGC-CGAC--------
Cairina moschata (EU755254)	--------------CT--GGGGCCACTACTAATCGC-AAGGCATGGC-CGAC--------
Ardea cinerea (Grey heron) bloodmeal (Sample B21)	--------------------ATCTACTACCC---TA-AAACGCTGGCTC-GC--------
Ardea cinerea (KJ190947)	------CCCTA---CT--GGATCTACTACCC---TA-AAACGCTGGCTC-GC--------
Passeriformes (Passerine bird) bloodmeal (Sample B19)	------CCC-A---CT--GGGTTCACTGCTA-CATA-AGCGACTGGT-CTGT--------
Pseudonestor xanthophrys (KM078809)	------CCC-A---CT--GGGTTCACTGTTA-CATA-AGACACTGGT-CTGC--------
Passeriformes (Passerine bird) bloodmeal (Sample B152)	------CCC-A---CT--GGGTATACGCACA-CACT-AACCGCTGGC-CTGC--------
Ficedula parva (FJ465224)	------CCC-A---CT--GGGCCCACTTACA-CATA-AGCCACTGGC-CTGC--------
Weaver bird (Ploceus baglaflecht reichnowi) bloodmeal (Sample B221)	------CCC-A---CT--GGGTTCACTGCCACA-TA-AGTTACTGG-TCCGT--------
Small bird (Weaver bird) bloodmeal (Sample B209)	------CCC-A---CT--GGGTTCACTGCCACA-TA-AGCTACTGG-TTCGT--------
Ploceus baglaflecht (AY283898)	------CCC-A---CT--GGGTTCACTGCCGCA-TA-AGCCACTGG-TCCGT--------
Icteridae (Black bird) bloodmeal (Sample B228)	------CCC-A---TC--GGGCCCACTCCCCTAACA-AGTCATTGG-TCCAC--------
Curaeus curaeus (JX516070)	------CCC-A---CT--GGGTTCACTGACACA-TA-AGACTTTGG-TCTAC--------
Micropteropus pusillus (Fruit bat) bloodmeal (Sample B34)	---------------------AAA--TA--A--ACC--AACTTTTACT-GG---ACTAGC
Micropteropus pusillus (JN398183)	--------------------AAAA--TA--A--ACC--AACTTTTACT-GG---ACTAGC
Bos taurus (Cow) bloodmeal (Sample B25A)	--------------TTAAGGAA----TA--AC-A-----ACAATCTCCATG--AGTTGGT
Bos taurus (Cow) bloodmeal (Sample B146)	--------------TTAAGGAA----TA--AC-A-----ACAATCTCCATG--AGTTGGT
Bos taurus (KJ789953)	--------------TTAAGGAA----TA--AC-A-----ACAATCTCCATG--AGTTGGT
Canis lupus (Dog) bloodmeal (Sample B105)	-----------------AGGCA----TA--AC-ATA-ACACCATTATTATG--AGTTAGC
Canis lupus (KJ789955)	-----------------AGGCA----TA--AC-ATA-ACACCATTATTATG--AGTTAGC
Equus asinus (Donkey) bloodmeal (Sample B93)	-----AACC-----CTCAGGGA----CA--AC-A---AAACTTTTGAT-TG--AATCAGC
Equus asinus (KM881681)	-----AACC-----CTCAGGGA----CA--AC-A---AAACTTTTGAT-TG--AATCAGC
Equus asinus (AP012271)	-----AACC-----CTCAGGGA----CA--AC-A---AAACTTTTGAT-TG--AATCAGC
Capra hircus (Goat) bloodmeal (Sample B217)	----------------AAGGGA----TA--AC-A-----ACATCCTTTATG--GACTAGC
Capra hircus (KP195268)	----------------AAGGGA----TA--AC-A-----ACATCCTTTATG--GACTAGC
Homo sapiens (Human) bloodmeal (Sample B148)	-----AACC-----CACAGGTCCT--AA--AC-T-----ACCA--AACCTG--CATTAAA
Homo sapiens (KP218948)	-----AACC-----CACAGGTCCT--AA--AC-T-----ACCA--AACCTG--CATTAAA
Arvicanthis niloticus (Rat) bloodmeal (Sample B36)	TAACTTAAC-----CTAATGGGAC--CA--AC-AT----A-AAAAAAAATA--AGCTAGT
Arvicanthis niloticus (AF141228)	TAATATAAC-----CTAATGGGCC---A--AC-AT----A-AAAAAAAATA--AGCTAGA
Ovis aries (Sheep) bloodmeal (Sample B183)	--------------CCAAGGGA----TA--AC-A-----ACACTCCTTATG--AGTTAAC
Ovis aries (KF312238)	--------------CCAAGGGA----TA--AC-A-----ACACTCCTTATG—AGTTAAC

```
                                                                          61         70        80        90        100       110       120
                                                                          |          |         |         |         |         |         |
                    Bufo regularis (African toad) bloodmeal (sample B70)  AGTTTTTGGTTGGGGTGACCGCGGAGCATAACATAACCTCC-----AT----GCTGA--A
                                               Bufo regularis (AF220889)  AGTTTTTGGTTGGGGTGACCGCGGAGCATAACATAACCTCC-----AT----GCTGA--A
Ptychadena anchietae (Anchieta's ridged frog) bloodmeal (Sample B79)  AGTTTTGGGTTGGGGTGACCGCGGAGAATAACCTAACCTCC-----GC----AATGAAAA
                                         Ptychadena anchietae (GQ183598)  AGTTTTGGGTTGGGGTGACCGCGGAGAATAACCTAACCTCC-----GC----AATGAAAA
               Ptychadena nilotica (Grass frog) bloodmeal (Sample B31)  AGCTTTAGGTTGGGGTGACCGCGGAGAAAAACTTAACCTCC-----AT----AATGAAAA
                                          Ptychadena nilotica (DQ525928)  AGCTTTAGGTTGGGGTGACCGCGGAGAAAAACTTAACCTCC-----AT----AATGAAAA
                  Hirundo rustica (Barn swallow) bloodmeal (Sample B21)  TTTTTTCGGTTGGGGCGACCTTGGAGCAAAACAAAACCTCC-----A-----AACACT-A
                                              Hirundo rustica (AB042382)  TTTTTTCGGTTGGGGCGACCTTGGAGCAAAACAAAACCTCC-----A-----AACACT-A
 Urocolius macrourus (mousebird) bloodmeal (Sample B11,22,B20,B229)  ATTTTTCGGTTGGGGCGACCTTGGAGTAAAACAGATCCTCC-----A-----AAAACA-A
                                          Urocolius macrourus (AF173589)  ATTTTTCGGTTGGGGCGACCTTGGAGTAAAACAGATCCTCC-----A-----AAAACA-A
                    Bubulcus ibis (Cattle Egret) bloodmeal (sample B180)  ATTTTTCGGTTGGGGCGACCTTGGAGAAAAACAAATCCTCC-----A-----AAAACA-A
                                                Bubulcus ibis (KJ190945)  ATTTTTCGGTTGGGGCGACCTTGGAGAAAAACAAATCCTCC-----A-----AAAACA-A
                          Gallus gallus (Chicken) bloodmeal (Sample B19)  ATTTTTCGGTTGGGGCGACCTTGGAGAAAAAAAAATCCTCC-----A-----AACCCACA
                                                Gallus gallus (AY236430)  ATTTTTCGGTTGGGGCGACCTTGGAGAAAAAAAAATCCTCC-----A-----AACCCACA
                  Gallus gallus variant (Chicken) bloodmeal (Sample B15)  ATTTTTTGGTTGGGGCGACCTTGGAGAAAAGAAAATCCTCC-----A-----AACCTACA
                                      Francolinus pintadeanus (EU165707)  ATTTTTCGGTTGGGGCGACCTTGGAGAAAAGAAAATCCTCC-----A-----AACATAAA
                        Streptopelia turtur(Dove) bloodmeal (Sample B5)  ATTTTTCGGTTGGGGCGACCTTGGAGAAAAACAAATCCTCC-----A-----AAAACA-A
                                          Streptopelia turtur (KC984248)  ATTTTTCGGTTGGGGCGACCTTGGAGAAAAGTAAATCCTCC-----A-----AAAACA-A
                  Cairina moschata (Muscovy duck) bloodmeal (Sample B6)  ATTTTTCGGTTGGGGCGACCTTGGAGAAAAACAGACCCTCC-----A-----AAAACA-A
                                             Cairina moschata (EU755254)  ATTTTTCGGTTGGGGCGACCTTGGAGAAAAACAGACCCTCC-----A-----AAAACA-A
                      Ardea cinerea (Grey heron) bloodmeal (Sample B21)  ATTTTTCGGTTGGGGCGACCTTGGAGAAAAACGAATCCTCC-----A-----AAGACA-A
                                                Ardea cinerea (KJ190947)  ATTTTTCGGTTGGGGCGACCTTGGAGAAAAACGAATCCTCC-----A-----AAGACA-A
             Passeriformes (Passerine bird) bloodmeal (Sample B19)  GTTTTTCGGTTGGGGCGACCTTGGAGCAAAACAAAACCTCC-----A-----AAAATT-A
                                     Pseudonestor xanthophrys (KM078809)  GTTTTTCGGTTGGGGCGACCTTGGAGCAAAACAGAACCTCC-----A-----AAAATT-A
            Passeriformes (Passerine bird) bloodmeal (Sample B152)  ATTTTTCGGTTGGGGCGACCTTGGAGAAAAACAAATCCTCC-----A-----AAAATT-A
                                              Ficedula parva (FJ465224)  ATTTTTCGGTTGGGGCGACCTTGGAGAAAAACAAATCCTCC-----A-----AAAACT-A
  Weaver bird (Ploceus baglaflecht reichnowi) bloodmeal (Sample B221)  GTTTTTCGGTTGGGGCGACCTTGGAGCAAAACAAAACCTCC-----A-----AAAATT-G
                    Small bird (Weaver bird) bloodmeal (Sample B209)  GTTTTTCGGTTGGGGCGACCTTGGAGCAAAACAAAACCTCC-----A-----AAAATT-G
                                          Ploceus baglaflecht (AY283898)  GTTTTTCGGTTGGGGCGACCTTGGAGCAAAACAAAACCTCC-----A-----AAAATT-G
                      Icteridae (Black bird) bloodmeal (Sample B228)  GTTTTTCGGTTGGGGCGACCTTGGAGCAAAGCAAAACCTCC-----A-----AAAATT-G
                                              Curaeus curaeus (JX516070)  GTTTTTCGGTTGGGGCGACCTTGGAGCAAAACAAAACCTCC-----A-----AAAATT-G
            Micropteropus pusillus (Fruit bat) bloodmeal (Sample B34)  AA-TTTAGGTTGGGGTGACCTCGGAATATAAAACAACTCCCGAGTGA-C--ACAGTCTA-
                                       Micropteropus pusillus (JN398183)  AA-TTTAGGTTGGGGTGACCTCGGAATATAAAACAACTCCCGAGTGA-C--ACAGTCTA-
                              Bos taurus (Cow) bloodmeal (Sample B25A)  AG-TTTCGGTTGGGGTGACCTCGGAGAATAAAAAATCCTCCGAGCGATTTTAAAGACT-A
                              Bos taurus (Cow) bloodmeal (Sample B146)  AG-TTTCGGTTGGGGTGACCTCGGAGAATAAAAAATCCTCCGAGCGATTTTAAAGACT-A
                                                   Bos taurus (KJ789953)  AG-TTTCGGTTGGGGTGACCTCGGAGAATAAAAAATCCTCCGAGCGATTTTAAAGACT-A
                             Canis lupus (Dog) bloodmeal (Sample B105)  AA-TTTAGGTTGGGGTGACCTCGGAATATAAAAAAACTCCCGAGTGAT--TAAAATTTA-
                                                  Canis lupus (KJ789955)  AA-TTTAGGTTGGGGTGACCTCGGAATATAAAAAAACTCCCGAGTGAT--TAAAATTTA-
                         Equus asinus (Donkey) bloodmeal (Sample B93)  AA-TTTCGGTTGGGGTGACCTCGGAGAACAAAACAACCTCCGAGTGATTT--AAATCTA-
                                                 Equus asinus (KM881681)  AA-TTTCGGTTGGGGTGACCTCGGAGAACAAAACAACCTCCGAGTGATTT--AAATCTA-
                                                 Equus asinus (AP012271)  AA-TTTCGGTTGGGGTGACCTCGGAGAACAAAACAACCTCCGAGTGATTT--AAATCTA-
                          Capra hircus (Goat) bloodmeal (Sample B217)  AG-TTTTGGTTGGGGTGACCTCGGAGAACAAGAGATCCTCCGAGCGATTTTAAAGACTA-
                                                 Capra hircus (KP195268)  AG-TTTTGGTTGGGGTGACCTCGGAGAACAAGAGATCCTCCGAGCGATTTTAAAGACTA-
                         Homo sapiens (Human) bloodmeal (Sample B148)  AA-TTTCGGTTGGGGCGACCTCGGAGCAGAACCCAACCTCCGAGCAGT---ACATGCTAA
                                                 Homo sapiens (KP218948)  AA-TTTCGGTTGGGGCGACCTCGGAGCAGAACCCAACCTCCGAGCAG---TACATGCTAA
                  Arvicanthis niloticus (Rat) bloodmeal (Sample B36)  AA-TTTCGGTTGGGGTGACCTCGGAGAATAAAAAATCCTCCGAATGATTAT--AGTTCA-
                                        Arvicanthis niloticus (AF141228)  AA-TTTCGGTTGGGGTGACCTCGGAGAATAAAAAATCCTCCGAATGATTTT--AGCATA-
                            Ovis aries (Sheep) bloodmeal (Sample B183)  AG-TTTCGGTTGGGGTGACCTCGGAGAACAGAAAATCCTCCGAGCGATTTTAAAGACTA-
                                                   Ovis aries (KF312238)  AG-TTTCGGTTGGGGTGACCTCGGAGAACAGAAAATCCTCCGAGCGATTTTAAAGACTA-
```

	121 130 140 150 160 170 180
Bufo regularis (African toad) bloodmeal (sample B70)	GGATTTAA-TT-CTAAGCTGAGACCTACGCCTCTAAGCATCAGCACACTGACATTAATTG
Bufo regularis (AF220889)	GGATTTAA-TT-CTAAGCTGAGACCTACGCCTCTAAGCATCAGCACACTGACATTAATTG
Ptychadena anchietae (Anchieta's ridged frog) bloodmeal (Sample B79)	GAATAATA-TC-CTAATCTAAGAGGGACACCTCTAAGAATTAATAAATTAACGTATGATG
Ptychadena anchietae (GQ183598)	GAATAATA-TC-CTAATCTAAGAGGGACACCTCTAAGAATTAATAAATTAACGTATGATG
Ptychadena nilotica (Grass frog) bloodmeal (Sample B31)	GAATAAAA-TC-CTAATCTATGAGCCACACCTCTAAGAATCAACAAATTGACATAAAATG
Ptychadena nilotica (DQ525928)	GAATAAAA-TC-CTAATCTATGAGCCACACCTCTAAGAATCAACAAATTGACATAAAATG
Hirundo rustica (Barn swallow) bloodmeal (Sample B21)	GACCACAC--ATCTAGACCAAGAGCTACAACTC----GACGTGCAAATAGC-ACCCA--G
Hirundo rustica (AB042382)	GACCACAC--ATCTAGACCAAGAGCTACGACTC----GACGTGCAAATAGC-ACCCA--G
Urocolius macrourus (mousebird) bloodmeal (Sample B11,22,B20,B229)	GACCT-AC-CCTCTTAATCAAGAGCCACCTCTC----AACATGCCAATAGCCA-CCA--G
Urocolius macrourus (AF173589)	GACCT-AC-CCTCTTAATCAAGAGCCACCTCTC----AACATGCCAATAGCCA-CCA--G
Bubulcus ibis (Cattle Egret) bloodmeal (sample B180)	GACCACAC--CTCTTAACCAAGAGCGACATCTC----TACGTGCTAACAGTAA-CCA--G
Bubulcus ibis (KJ190945)	GACCACAC--CTCTTAACCAAGAGCGACATCTC----TACGTGCTAACAGTAA-CCA--G
Gallus gallus (Chicken) bloodmeal (Sample B19)	GACCACAA--CTCTTCACTAAGACCAACTCCTC----AAAGTACCAACAGTAA-CCA--G
Gallus gallus (AY236430)	GACCACAA--CTCTTCACTAAGACCAACTCCTC----AAAGTACCAACAGTAA-CCA--G
Gallus gallus variant (Chicken) bloodmeal (Sample B15)	GACCACAA--CTCTTCACTAAGACCAACCCTTC----AAAGTACAAATAGTAA-CTA--G
Francolinus pintadeanus (EU165707)	GACCACAA--CTCTTTACCAAGACCAACACCTC----AAAGTACTAATAGTAATTTA--G
Streptopelia turtur(Dove) bloodmeal (Sample B5)	GACCACCC-CCTCTTAACTAAGAGCAACCCCTC----AACGTACTAATAGTAA-CCA--G
Streptopelia turtur (KC984248)	GACCACTC-CCTCTTAACTAAGAGCAACCCCTC----AACGTACTAACAGTAA-CCA--G
Cairina moschata (Muscovy duck) bloodmeal (Sample B6)	GACCACAC--CTCTTTACTTAGAGCCACCCCTC----AAAGTGCTAATAGCGA-CCA--G
Cairina moschata (EU755254)	GACCACAC--CTCTTTACTTAGAGCCACCCCTC----AAAGTGCTAATAGCGA-CCA--G
Ardea cinerea (Grey heron) bloodmeal (Sample B21)	GACCACAC--CTCTTAACCAAGAGCAACATCTC----TACGTGCTAATAGTAA-CCA--G
Ardea cinerea (KJ190947)	GACCACAC--CTCTTAACCAAGAGCAACATCTC----TACGTGCTAATAGTAA-CCA--G
Passeriformes (Passerine bird) bloodmeal (Sample B19)	GACCACAC--CTCTAGACTAAGAGCAACCCCTC----AACGTGCTAATAGCAA-CCA--G
Pseudonestor xanthophrys (KM078809)	GACCACAC--CTCTAGACTGAGAGCAACCCCTC----AACGTGCTAATAGCAC-CCA--G
Passeriformes (Passerine bird) bloodmeal (Sample B152)	GACCACAC--CTCTAGACTAAGAGCTACCCCTC----AACGTGCTAATAGCAA-CCA--G
Ficedula parva (FJ465224)	GACCACAC--CTCTAGACTAAGAGCAACCCCTC----AACGTGCTAATAGCAA-CCA--G
Weaver bird (Ploceus baglaflecht reichnowi) bloodmeal (Sample B221)	GACCATAC--CTCCAGACCAAGAGCAACCCCTC----AACGTGCTAATAGC-ATCCA--G
Small bird (Weaver bird) bloodmeal (Sample B209)	GACCACAA--CTCCAGACCAAGAGCAACCTCTC----AACGTGCTAATAGC-ATCCA--G
Ploceus baglaflecht (AY283898)	GATCACAC--CTCCAGACCAAGAGCAACCCCTC----AACGTGCTAATAGC-ATCCA--G
Icteridae (Black bird) bloodmeal (Sample B228)	GACCACAC--CTCCAGACCAAGAGCAACCACTC----AACGTGCTAATAGC-AACCA--G
Curaeus curaeus (JX516070)	GACCATAC--CTCTAGACTAAGAGCAACCTCTC----AACGTGCTAATAGC-AACCA--G
Micropteropus pusillus (Fruit bat) bloodmeal (Sample B34)	GACT-AACAAGTCGAAC--CCT-CTATCA--TT-----------CA-GTG--ATCCAA--
Micropteropus pusillus (JN398183)	GACT-AACAAGTCGAAC--CCT-CTATCA--TT-----------CA-GTG--ATCCAA--
Bos taurus (Cow) bloodmeal (Sample B25A)	GACC-CACAAGTCAAATC--ACT-CTATCGCTC-------------ATTG--ATCCAA--
Bos taurus (Cow) bloodmeal (Sample B146)	GACC-CACAAGTCAAATT--ACT-CTATCGCTC-------------ATTG--ATCCAA--
Bos taurus (KJ789953)	GACC-CACAAGTCAAATC--ACT-CTATCGCTC-------------ATTG--ATCCAA--
Canis lupus (Dog) bloodmeal (Sample B105)	GACC-CACAAGTCAAAAT-A-CA-ACATCA--CT----------T-ATTG--ATCCAA--
Canis lupus (KJ789955)	GACC-CACAAGTCAAAAT--ACA-ACATCA--CT----------T-ATTG--ATCCAA--
Equus asinus (Donkey) bloodmeal (Sample B93)	GACT-AACCAGTCAAAAT--ACA-TAATCA--CT----------TA-TTG--ATCCAA--
Equus asinus (KM881681)	GACT-AACCAGTCAAAAT--ACA-TAATCA--CT----------TA-TTG--ATCCAA--
Equus asinus (AP012271)	GACT-AACCAGTCAAAAT--ACA-TAATCA--CT----------TA-TTG--ATCCAA--
Capra hircus (Goat) bloodmeal (Sample B217)	GACTT-ACAAGTCAAATC-AA-A-TTATCG--CT----------TA-TTG--ATCCAA--
Capra hircus (KP195268)	GACTT-ACAAGTCAAATC-AA-A-TTATCG--CT----------TA-TTG--ATCCAA--
Homo sapiens (Human) bloodmeal (Sample B148)	GACTTCACCAGTCAAAGC-GA-A-CTACTATACT----------CAATTG--ATCCAA--
Homo sapiens (KP218948)	GACTTCACCAGTCAAAGC-GA-A-CTACTATACT----------CAATTG--ATCCAA--
Arvicanthis niloticus (Rat) bloodmeal (Sample B36)	GACC-AACAAGTCAAAAC-AACA-CTTTAAATCT----------TA-TTG--ATCCAA--
Arvicanthis niloticus (AF141228)	GACT-AACAAGTCAAAGC-AACA-TTACAAATCT----------TA-TTG--ACCCAA--
Ovis aries (Sheep) bloodmeal (Sample B183)	GACT-AACAAGTCAAACC-AA-A-CCATCG--CT----------TA-TTG--ATCCAA--
Ovis aries (KF312238)	GACT-AACAAGTCAAACC-AA-A-CCATCG--CT----------TA-TTG--ATCCAA—

```
                                                                    181       190        200        210
                                                                     |         |          |          |
Bufo regularis (Leopard toad) bloodmeal (sample B70)                ACCCAATAC----AATTGAGCAACGAACCAAGTTACCC
Bufo regularis (AF220889)                                           ACCCAATAC----AATTGAGCAACGAACCAAGTTACCC
Ptychadena anchietae (Anchieta's ridged frog) bloodmeal (Sample B79) ATCCAATACTTATATTTGATCAATGAACCAAGTTACCC
Ptychadena anchietae (GQ183598)                                     ATCCAATACTTATATTTGATCAATGAACCAAGTTACCC
Ptychadena nilotica (Grass frog) bloodmeal (Sample B31)             ACCCGATA-----ATTCGATCAATGAACCAAGTTACCC
Ptychadena nilotica (DQ525928)                                      ACCCGATA-----ATTCGATCAATGAACCAAGTTACCC
Hirundo rustica (Barn swallow) bloodmeal (Sample B21)               ACCCAATAA----AATTGATCAATGGACCAAGCTACCC
Hirundo rustica (AB042382)                                          ACCCAATAA----AATTGATCAATGGACCAAGCTACCC
Urocolius macrourus (mousebird) bloodmeal (Sample B11,22,B20,B229)  ACCCAATAC----ACTTGATTAATGGACCAAGCTACCC
Urocolius macrourus (AF173589)                                      ACCCAATAC----ACTTGATTAATGGACCAAGCTACCC
Bubulcus ibis (Cattle Egret) bloodmeal (sample B180)                ACCCAATAT----AATTGATTAATGAACCAAGCTACCC
Bubulcus ibis (KJ190945)                                            ACCCAATAT----AATTGATTAATGAACCAAGCTACCC
Gallus gallus (Chicken) bloodmeal (Sample B19)                      ACCCAATAT----AATTGAGCAATGGACCAAGCTACCC
Gallus gallus (AY236430)                                            ACCCAATAT----AATTGAGCAATGGACCAAGCTACCC
Gallus gallus variant (Chicken) bloodmeal (Sample B15)              ACCCAATAC----AATTGATCAATGGACCAAGCTACCC
Francolinus pintadeanus (EU165707)                                  ACCCAATAC----AATTGATCAATGGACCAAGCTACCC
Streptopelia turtur(Dove) bloodmeal (Sample B5)                     ACCCAATAC----AATTGACCAATGGACCAAGCTACCC
Streptopelia turtur (KC984248)                                      ACCCAATAC----AATTGATCAATGGACCAAGCTACCC
Cairina moschata (Muscovy duck) bloodmeal (Sample B6)               ACCCAATAT----AATTGATTAATGGACCAAGCTACCC
Cairina moschata (EU755254)                                         ACCCAATAT----AATTGATTAATGGACCAAGCTACCC
Ardea cinerea (Grey heron) bloodmeal (Sample B21)                   ACCCAATAC----AATTGATTAATGAACCAAGCTACCC
Ardea cinerea (KJ190947)                                            ACCCAATAC----AATTGATTAATGAACCAAGCTACCC
Passeriformes (Passerine bird) bloodmeal (Sample B19)               ACCCAATAT----AATTGATCAATGGACCAAGCTACCC
Pseudonestor xanthophrys (KM078809)                                 ACCCAATAT----AATTGATCAATGGACCAAGCTACCC
Passeriformes (Passerine bird) bloodmeal (Sample B152)              ACCCAATAT----AATTGATCAATGGACCAAGCTACCC
Ficedula parva (FJ465224)                                           ACCCAATAT----AATTGATCAATGGACCAAGCTACCC
Weaver bird (Ploceus baglaflecht reichnowi) bloodmeal (Sample B221) ACCCAATAC----AATTGATCAATGGACCAAGCTACCC
Small bird (Weaver bird) bloodmeal (Sample B209)                    ACCCAATAC----AATTGATCAATGGACCAAGCTACCC
Ploceus baglaflecht (AY283898)                                      ACCCAATAC----AATTGACCAATGGACCAAGCTACCC
Icteridae (Black bird) bloodmeal (Sample B228)                      ATCCAATAT----AATTGATCAATGGACCAAGCTACCC
Curaeus curaeus (JX516070)                                          ACCCAATAC----AATTGATCAATGGACCAAGCTACCC
Micropteropus pusillus (Fruit bat) bloodmeal (Sample B34)           ----TTT-CTT----TTGATCAACGGAACAAGTTACCC
Micropteropus pusillus (JN398183)                                   ----TTT-CTT----TTGATCAACGGAACAAGT-----
Bos taurus (Cow) bloodmeal (Sample B25A)                            ----AAA---C----TTGATCAACGGAACAAGT-----
Bos taurus (Cow) bloodmeal (Sample B146)                            ----AAA---C----TTGATCAACGGAACAAGT-----
Bos taurus (KJ789953)                                               ----AAA---C----TTGATCAACGGAACAAGT-----
Canis lupus (Dog) bloodmeal (Sample B105)                           ----TAA-TTT----TTGATCAACGGAACAAGT-----
Canis lupus (KJ789955)                                              ----TAA-TTT----TTGATCAACGGAACAAGTT----
Equus asinus (Donkey) bloodmeal (Sample B93)                        ----ACC---T----TTGATCAACGGAATAAGT-----
Equus asinus (KM881681)                                             ----ACC---T----TTGATCAACGGAACAAGT-----
Equus asinus (AP012271)                                             ----ACC---T----TTGATCAACGGAACAAGT-----
Capra hircus (Goat) bloodmeal (Sample B217)                         ----AAA--AC----TTGATCAACGGAACAAGT-----
Capra hircus (KP195268)                                             ----AAA--AC----TTGATCAACGGAACAAGT-----
Homo sapiens (Human) bloodmeal (Sample B148)                        ----TAA-C------TTGACCAACGGAACAAGT-----
Homo sapiens (KP218948)                                             ----TAA-C------TTGACCAACGGAACAAGT-----
Arvicanthis niloticus (Rat) bloodmeal (Sample B36)                  ----AACTA-T----TTGATCAACGGACCAAGTTACCC
Arvicanthis niloticus (AF141228)                                    ----ATCTATT----TTGATCAACGGACCAAGTTACCC
Ovis aries (Sheep) bloodmeal (Sample B183)                          ----AAA---C----TTGATCAACGGAACAAGT-----
Ovis aries (KF312238)                                               ----AAA---C----TTGATCAACGGAACAAGTT----
```
